# Thromboembolic Events in Patients Undergoing Neoadjuvant Chemotherapy and Radical Cystectomy for Muscle-Invasive Bladder Cancer: A Study of Renal Impairment in Relation to Potential Thromboprophylaxis

**DOI:** 10.3390/jpm12121961

**Published:** 2022-11-27

**Authors:** Harriet Rydell, Anna Ericson, Victoria Eriksson, Markus Johansson, Johan Svensson, Viqar Banday, Amir Sherif

**Affiliations:** 1 Department of Surgical and Perioperative Sciences, Urology and Andrology, Umeå University, 90187 Umeå, Sweden; 2 Department of Surgery, Urology Section, Sundsvall-Härnösand Hospital, 85186 Sundsvall, Sweden; 3 Department of Statistics, Umeå School of Business, Economics and Statistics, Umeå University, 90187 Umeå, Sweden

**Keywords:** complications, cystectomy, low-molecular-weight heparin, neoadjuvant therapy, thromboembolism, urinary bladder neoplasms

## Abstract

Recent studies on patients with muscle-invasive bladder cancer (MIBC) undergoing neoadjuvant chemotherapy (NAC) have shown an association between NAC and thromboembolic events (TEE) prior to radical cystectomy (RC). Recent studies suggest that central venous access catheters (CVAs) may induce TEEs, and low-molecular-weight heparin (LMWH) has been mentioned as possible prophylaxis. However, other studies have shown a high incidence of decreased renal function in these patients. The purpose of this study was to determine the portion of MIBC patients with NAC-induced TEEs who had decreased preoperative renal function for whom LMWH potentially would not be beneficial as prophylaxis. We identified 459 cystectomized MIBC patients from two Swedish medical centers from 2009 to 2021. The inclusion criterion of cT2-T4aN0M0 resulted in 220 eligible patients, who were further divided into NAC-administered (n = 187) and NAC-eligible (n = 33), the tentative control group. Values of renal function before, during, and after each NAC cycle were retrospectively collected from individual medical records. Amongst the NAC-administered patients with TEE (n = 29), 41% (95% CI 23.5–61.1%) of patients had decreased renal function. Thus, a substantial portion of NAC-administered patients who developed TEEs had reduced renal function and would have been less likely to have benefited from renal clearance-dependent LMWH prophylaxis.

## 1. Introduction

Urinary bladder cancer accounted for around 570,000 new cancer cases and about 200,000 deaths worldwide in 2020, making it the tenth most commonly diagnosed cancer [1]. Bladder cancer manifests either as non-muscle-invasive cancer or a muscle-invasive form (MIBC); the latter form accounts for approximately 25% of cases at diagnosis. Urothelial MIBC was, prior to the introduction of neoadjuvant chemotherapy (NAC), associated with a poor prognosis, with a 5-year overall survival (OS) post-RC of 50%. The current treatment regimen in Europe for MIBC includes NAC in medically fit patients and radical cystectomy (RC) [2]. The inclusion of NAC pre-RC aims to eradicate micro-metastatic dissemination and has been shown to significantly increase survival compared with RC only [3,4]. We previously showed that chemo-responding patients who downstaged with complete response (CR) to pT0N0M0) had an absolute risk reduction of 31% for death at the five-year median observation time (OS) [5]. 

The NAC regimen for MIBC patients in Sweden in most centers consists of a cisplatin-based combination of methotrexate, vinblastine, doxorubicin, and cisplatin (MVAC), while in patients who are ineligible for cisplatin, a carboplatin-gemcitabine combination is used [6]. Eligibility for NAC, according to Swedish guidelines, are age ≤ 75 years, renal function with eGFR > 50–60, no hearing impediments, and acceptable comorbidities [7]. NAC treatment is generally well tolerated; however, adverse events, including thromboembolic events (TEEs). have been shown to occur during and after NAC treatment [8,9]. Eriksson et al., using a multicenter clinical dataset, showed serious grade 4 TEEs in patients treated with NAC. In addition, acute kidney injury and chronic kidney disease were seen in 41% and 11% of patients, respectively [10]. Interestingly, 30% of patients receiving NAC had to terminate the treatment prematurely. Among these patients, 62% of terminations were due to acute kidney injury. There was a significant association between decreased kidney injury and increased downstaging, possibly reflecting the effect of completed intended NAC cycles on downstaging, as reported earlier [5]. Renal impairment is a fraught consequence of cisplatin-based combinations and one of the key determinants for NAC eligibility [7,11]. Mehrazin et al., in a recent study, showed a continued decline in eGFR rates in MIBC-NAC patients after discharge. In their study, the rate of decline was related to eGFR rates at discharge, with up to 43% of the patients showing a decline [12]. Deep vein thrombosis (DVT) and pulmonary embolism (PE) are two lethal complications during NAC and RC. Endothelial damage and the hypercoagulable state during cancer treatment continues post treatment [13]. Thromboprophylaxis pre- and post-surgery has become a standard in tertiary care centers and is highly encouraged as a quality-of-care indicator [14]. Low-molecular-weight heparin (LMWH) is frequently prescribed for the prevention and treatment of TEEs in patients [15]. LMWH provides a reduction in the incidence of venous thromboembolism and requires minimal monitoring [16,17]. However, combined with impaired renal function, the major mode of excretion for LMWH, there is an increased risk of supratherapeutic accumulation of LMWH with an associated risk of major bleeding [12]. To the best of our knowledge, no international or national guidelines exist advocating LMWH as prophylaxis against TEEs amongst MIBC-NAC patients during the NAC period. In this retrospective study, we investigate the incidence of NAC-induced renal impairment in our research database. The objective was to assess the incidence of patients with NAC-induced TEEs who may not have benefited from LMWH if prophylaxis had hypothetically been used.

## 2. Materials and Methods

### 2.1. Data Collection and NAC Eligibility

**Figure 1:**
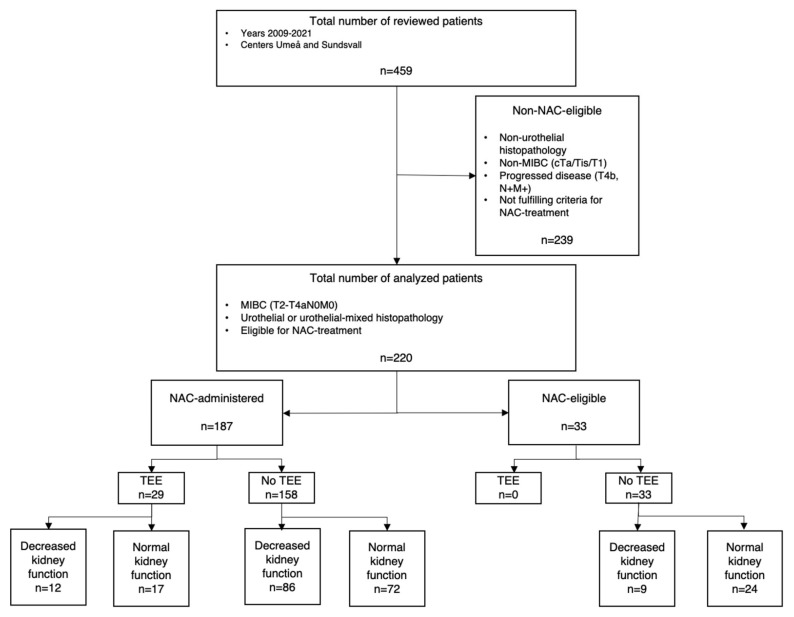
Flowchart of study population. MIBC; muscle-invasive bladder cancer. NAC; neoadjuvant chemotherapy. TEE; thromboembolic event.

The study population was derived from an extensive dataset based on records of radically cystectomized patients with urothelial carcinoma in the urinary bladder in the years 2009–2021. Patients’ data were retrospectively collected from individual medical records from the two participating Swedish centers: Norrlands Universitetsjukhus in Umeå and Länssjukhuset in Sundsvall. The inclusion criteria allowed for MIBC carcinomas categorized as cT2-T4bN0M0 with urothelial or mixed-type histopathology, thus excluding non-muscle-invasive cancers, disseminated disease before treatment initiation, and histopathology without any urothelial component. Hence, of the 459 radically cystectomized patients who were initially available for consideration, 220 were suitable for analysis, as 239 patients were excluded due to unfit histopathology, non-cT2-4aN0M0, or not fulfilling the criteria for NAC treatment and thus being deemed as non-NAC-eligible patients. For detailed information on patient selection, see Figure 1. 

The majority of MIBC patients in Sweden today receive 3 or 4 cycles of chemotherapy before RC, and in 2021, 71% of all MIBC patients received NAC in Umeå. On the contrary, in 2009, only 17% received NAC in Umeå, as NAC was not recommended by clinical guidelines until more recent years. Hence, a portion of all MIBC patients in Sweden did not receive NAC and was solely radically cystectomized. As chemotherapy by time was introduced into modern MIBC guidelines, the number of NAC-administrated patients rose. However, a few patients still are untreated due to other circumstances; for example, some choose to reject NAC. Thus, non-NAC but NAC-eligible patients provide us with a suitable parallel control group, as these patients can be regarded as exhibiting the same clinical status as those treated with NAC (Table 1). Consequently, this study focused on the NAC-administered patients (n = 187) and NAC-eligible non-NAC patients (n = 33), who were considered being the tentative control group. By analyzing the NAC-eligible patients, any confounding factors regarding the carcinoma and its innate effects on the cohort’s kidney function could be overviewed. The basic characteristics of both NAC-treated as well as NAC-eligible patients are shown in Table 1. To establish which patients would have been eligible for NAC, medical journals and preestablished NAC criteria were used with the guidance of a senior urologist and the national guidelines. To be considered NAC-eligible, patients had to be ≤75 years old with an eGFR > 50 or creatinine < 100 and a Charlson Age Comorbidity Index (CACI) of 6 or less. The CACI is used to determine a standardized 10-year survival prediction and was individually calculated for each patient. If a patient fulfilled the criteria to receive NAC but the multidisciplinary team recommended abstaining from treatment, the latter judgement was regarded by the study being the final verdict regarding a patient’s NAC eligibility.

Laboratory values regarding renal function were gathered, adding to the database’s already large collection of clinicopathological variables, such as age, gender, smoking habits, BMI, ASA, CACI, and TEE. Values for renal function are monitored in MIBC patients but are rarely measured by one single variable. Thus, four measurements were obtained for the study to obtain a conclusive portrayal of each patient’s clinical kidney status: serum/plasma creatinine (S/P-creatinine), eGFR by cystatin c, eGFR by creatinine and chrome-ethylenediaminetetraacetic acid (Cr-EDTA). The cut-offs for each measurement were in accordance with Swedish clinical practice and the standardized reference intervals: S/P-creatinine > 90 for females and > 100 for males, eGFR (creatinine) < 60, eGFR (cystatin c) < 60, and Cr-EDTA < 60. The reference intervals for all analyzed measurements are presented in Table 2. Values were collected at predetermined time intervals: pre-TUR-b, before initiation of NAC, after each provided NAC cycle, and, finally, before RC. Not all patients had an available value for each measurement in every time period, and if values were inaccessible, they were considered missing data. As no conclusion can be made from unavailable measurements, the study considered missing data to be non-pathological values. No patient had missing values in all 4 extracted measurements. Utilizing each exact value for every available measurement that was collected allowed the stratification of the cohort’s population into two sub-categories: patients with *or* without reduced kidney function. As one patient could potentially have either one single or several pathological values, multiple groupings regarding the type of measurement of kidney function at different time periods were considered.

Another distinction in the study cohort was made by separating the NAC-administrated who did or did not suffer a TEE. The TEE incidences, presented in Table 3, were used for analysis, with PE, DVT, TEEs anatomically related to the central venous access catheters (CVA), angina/myocardial infarction (MI), and transient ischemic attack (TIA)/stroke used as included types of events. The events were registered if the time of the TEE occurred during the period from TUR-b to RC. Four patients had a total number of 2 TEEs, and one patient had a total of 3 TEEs.

### 2.2. Statistics

Population characteristics for interval variables are described as means and standard deviation, and those for nominal variables are described as frequencies and percentages. The confidence interval for the percentage of patients with reduced kidney function among the defined groups of the cohort was calculated with the Clopper–Pearson method. The statistical analyses were performed with IBM SPSS Statistics for Windows, Version 28.0. Armonk, NY, USA: IBM Corp.

### 2.3. Ethics

The study was approved by the regional ethics board in Umeå: EPN-Umeå, dnr: 2013/463-31M and amendment 2016/403-32. The study conformed to the provisions of the Declaration of Helsinki (as revised in Fortaleza, Brazil, October 2013). The regional ethics board specifically decided that informed consent from the participants was to be considered redundant, especially due to the high mortality of MIBC as well as the retrospective nature of the study.

## 3. Results

The clinicopathological variables for each of the 220 analyzed patients were extracted from the extensive official database charting all MIBC patients who have undergone RC in Sweden and are presented in Table 1. Amongst these variables, TEE incidences were collected, enabling division into subgroups depending on whether patients suffered postulated NAC-induced thrombosis or not. Twenty-nine patients in the NAC-administrated group suffered one or more TEEs. Out of them, 18 were PEs and 11 were thromboses anatomically connected to the CVA. Three patients suffered thrombophlebitis as a TEE, two had DVTs, and, finally, one patient presented with angina. Four patients developed two individual TEEs, and one patient developed three. 

The findings regarding pathological renal values before the initiation of chemotherapy are presented in Table 4, elucidating any existing kidney damage in the study population pre-NAC and thus establishing the baseline measurements in each group. Non-pathological values for P/S-creatinine and eGFR are predetermined requirements for NAC eligibility; consequently, no patients in this group had reduced kidney function. In addition, as no patient in the NAC-eligible group suffered any TEE pre-RC, only patients without TEEs are presented. The reduced kidney function in the study population during the entire period, TUR-b to RC, is presented in Table 5. In the NAC-administrated group, 98 patients (52%) out of the total number of 187 had reduced kidney function pre-RC. Pathological measurements were found in 12 patients belonging to the NAC-administrated subgroup who also suffered TEEs (n = 29), resulting in 41% (95% CI 23–61%). 

Table 6 presents the portion of the study population in which at least one of the four predetermined and analyzed measurements were accessible. Out of the 220 patients, 35 (16%) had no obtainable values between TUR-b and the first NAC cycle and were thus left as missing data.

## 4. Discussion

According to our findings, a considerable portion of the NAC-administrated patients who suffered a TEE during treatment had reduced renal function, as 41% (95% CI 23–61%) had one or more pathological values throughout the entire time period, up until the day of cystectomy. Fifty-four percent of the NAC-administrated patients who underwent their chemotherapy without a thrombolytic adverse event also had reduced kidney function, presenting a similar result as the TEE group. The most frequent pathological value amongst the NAC-treated TEE patients was P/S-creatinine, with nine patients (31%) showcasing a higher value than the established reference interval. This is possibly in accordance with P/S-creatinine being the most frequently used measurement for kidney function in Sweden. No noteworthy differences were found between the NAC-administrated subgroups regarding kidney function before the initiation of chemotherapy. Hence, the two groups of TEE patients and those who did not suffer a TEE could be considered to have the same baseline renal function before the first NAC cycle. 

The main objective of the study was to investigate renal function in NAC-administrated patients who had suffered a TEE and to assess their ability to potentially respond to LMWH-prophylaxis. Hence, the participants were divided into subgroups of those who did or did not develop thrombosis during the time between diagnosis and RC. NAC inherently contributes to an amplified TEE risk in MIBC patients undergoing RC, and recent findings have suggested that the choice of CVA for chemotherapy can influence the increased risk [8,18,19]. As LMWH regularly is used in TEE treatment and commonly as prophylaxis in other high-risk patient groups, suggestions have been made to also use LMWH in cases in which MIBC-NAC patients would be considered high-risk for TEE. Accordingly, Mehrazin et al. suggested recently that established reduced kidney function would render heparin-based prophylaxis sub-therapeutic by accumulation, which in turn would increase the risk of clinically significant bleeding. Our results showed that in NAC-administrated patients who did suffer a TEE, 41% would potentially not have responded to prophylactic treatment. Thus, the argument can be made to contemplate whether LMWH can be considered the single solution to the increased TEE risk in this patient group or whether instead a shift in the main focus should be made to the choice of CVA. 

As the hypothetical LMWH treatment would be initiated at the start of chemotherapy (NAC), the distinction between the TEE and non-TEE patients would not be yet established. Therefore, there is relevance in also establishing the incidence of reduced renal function in NAC-administrated patients who have not suffered a TEE. In the NAC-administrated no-TEE group, 54% were found to have reduced renal function during the entire pre-RC period. Hence, this group showed similar results to the TEE-positive subgroup, indicating that LMWH could be subtherapeutic in the NAC-MIBC group as a whole. The NAC-eligible non-NAC group presented with a lower prevalence of pathological renal values than the groups (w/wo TEE) treated with NAC, possibly reflecting the negative effect that MIBC might inherently possess on kidney function. 

However, because of this study’s numerically limited cohort population, a larger patient selection is needed to further establish the incidence of reduced kidney function amongst NAC-administrated MIBC patients. Further important associated subjects to evaluate are how the current clinical use of LMWH contributes to reduced renal function, as well as extending the time period of analysis both pre-RC as well as post-RC. A strength of this study is that the analyzed measurements are meticulously extracted from individual medical records; thus, the presented findings could be regarded as highly reliable. 

## 5. Conclusions

Our findings show that a substantial portion of NAC-administered patients with TEEs had reduced renal function pre-RC, and therefore, they would potentially not be suited for renal clearance-dependent LMWH prophylaxis. However, further investigations are needed to fully comprehend the prevalence of reduced renal function amongst MIBC patients. 

## Figures and Tables

**Table 1:** Baseline characteristics of the study cohorts.

		NAC-ADMINISTRATED n = 187			NAC-ELIGIBLE n = 33
		WITH TEE n = 29	NO TEE n = 158	TEE + NO TEE n = 187	
VARIABLE		Mean (SD)	Mean (SD)	Mean (SD)	Mean (SD)
AGE		67 (7)	68 (7)	68 (7)	67 (9)
CACI		5 (1)	5 (1)	5 (1)	5 (1)
NO. NAC CYCLES		3 (1)	3 (1)	3 (1)	0 (0)
		n (%)	n (%)	n (%)	n (%)
MEDICAL CENTER					
	UMEÅ	25 (86)	120 (76)	145 (77)	22 (67)
	SUNDSVALL	4 (14)	38 (24)	42 (22)	11 (33)
GENDER					
	WOMAN	4 (14)	41 (26)	45 (24)	5 (15)
	MAN	25 (86)	118 (75)	143 (76)	28 (85)
cT-STADIUM					
	T2	15 (51)	91 (57)	106 (57)	22 (67)
	T3	12 (41)	54 (34)	66 (35)	11 (33)
	T4 & T4a	2 (7)	13 (8)	15 (8)	0 (0)
THROMBOSIS PROPHYLAXIS					
	Anticoagulant	12 (41)	9 (6)	21 (11)	3 (9)
	Antiplatelet	28 (96)	23 (14)	51 (27)	5 (15)

NAC: neoadjuvant chemotherapy. TEE: thromboembolic event. SD: standard deviation. CACI: Charlson comorbidity index.

**Table 2:** Reference interval and cut-off values for pathology of included measurements.

MEASUREMENT		REFERENCE INTERVAL	CUT OFF VALUE
**P/S-Creatinine**			
	Male	60–100	>100
	Female	50–90	>90
**eGFR (creatinine)**			
	18–50 years	>80	<60
	>50 years	>60	<60
**eGFR (cystatin c)**			
	18–50 years	>80	<60
	>50 years	>60	<60
**Cr-EDTA**			
	20–50 years	80–125 mL/min/1.73 m^2^	<60
	51–65 years	60–110 mL/min/1.73 m^2^	<60
	66–80 years	50–90 mL/min/1.73 m^2^	<60

P/S: plasma or serum. eGFR: estimated glomerular filtration rate. Cr-EDTA: chrome ethylenediaminetetraacetic acid.

**Table 3:** TEE incidences amongst NAC-administrated patients pre-cystectomy.

	NAC-ADMINISTRATED n = 187
	WITH TEE n = 29
**TYPE OF TEE**	
**DVT**	2
**THROMBOPHLEBITIS**	3
**PE**	18
**FROM CVA**	11
**ANGINA/MI**	1

TEE, thromboembolic events; NAC, neoadjuvant chemotherapy; DVT, deep vein thrombosis; PE, pulmonary embolism; FROM CVA, anatomically related to the central venous access; MI, myocardial infarction.

**Table 4:** Reduced kidney function after TUR-B, before first NAC cycle.

			NAC-ADMINISTRATED (n = 187)			NAC-ELIBIGLE (n = 33)
			WITH TEE n (%)	NO TEE n (%)	TEE + NO TEE n (%)	NO TEE n (%)
			n = 29	n = 158	n = 187	n = 33
**REDUCED KIDNEY FUNCTION PRE-NAC 1**						
**MEASUREMENT**						
	P/S-Creatinine		6 (21)	29 (18)	35 (19)	0 (0)
	eGFR (creatinine)		3 (10)	14 (9)	17 (9)	0 (0)
	eGFR (cystatin c)		1 (3)	25 (16)	26 (14)	0 (0)
	Cr-EDTA		3 (10)	21 (13)	24 (13)	0 (0)
**ALL MEASUREMENTS**						
			9 (31)	50 (32)	59 (31)	0 (0)

For NAC eligibility, non-pathological renal functional values were required as predetermined by the study; thus, no NAC-eligible patients present any kidney damage at diagnosis. All measurements: one or more of the four values were pathological in the same patient. TUR-B: transurethral resection of the bladder. NAC: neoadjuvant chemotherapy. TEE: thromboembolic event. P/S: plasma or serum. eGFR: estimated glomerular filtration rate. Cr-EDTA: chrome ethylenediaminetetraacetic acid.

**Table 5:** Reduced kidney function at any point during the entire period; pre-TUR-B to before cystectomy.

			NAC-ADMINISTRATED n = 187			NAC-ELIBIGLE (n = 33)
			WITH TEE n (%)	NO TEE n (%)	TEE + NO TEE n (%)	NO TEE n (%)
			n = 29	n = 158	n = 187	n = 33
**REDUCED KIDNEY FUNCTION PRE CE**						
**MEASUREMENT**						
	P/S-Creatinine		9 (31)	69 (44)	78 (42)	9 (27)
	eGFR (creatinine)		5 (17)	37 (23)	42 (22)	1 (3)
	eGFR (cystatin c)		2 (7)	31 (20)	33 (18)	0 (0)
	Cr-EDTA		3 (10)	22 (14)	25 (13)	0 (0)
**ALL MEASUREMENTS**						
			12 (41)	86 (54)	98 (52)	9 (27)

All measurements: one or more of the four values were pathological in the same patient. TUR-B: Transurethral resection of the bladder. NAC: neoadjuvant chemotherapy. TEE: thromboembolic event. RC: radical cystectomy. P/S: plasma or serum. eGFR: estimated glomerular filtration rate. Cr-EDTA: chrome ethylenediaminetetraacetic acid.

**Table 6:** Patients with available values at any point in the entire time period: TUR-b–pre-cystectomy.

		NAC-ADMINISTRATED n = 187			NAC-ELIGIBLE n = 33
		WITH TEE n (%)	NO TEE n (%)	TEE + NO TEE n (%)	NO TEE n (%)
		n = 29	n = 158	n = 187	n = 33
**MEASUREMENT**					
	P/S-Creatinine	29 (100)	158 (100)	187 (100)	32 (97)
	eGFR (creatinine)	20 (69)	109 (69)	129 (69)	10 (30)
	eGFR (cystatin c)	0 (0)	15 (9)	15 (8)	1 (3)
	Cr-EDTA	1 (3)	2 (1)	3 (2)	0 (0)

TUR-b: transurethral resection of the bladder, NAC: neoadjuvant chemotherapy. TEE: thromboembolic event. P/S: plasma or serum. eGFR: estimated glomerular filtration rate. Cr-EDTA: chrome ethylenediaminetetraacetic acid.

## Data Availability

On reasonable request, the corresponding author can make available all codified data from the clinical database used for this study.
